# Dying with Dementia: Is Hospice the Answer?

**DOI:** 10.1093/geroni/igaf122.1107

**Published:** 2025-12-31

**Authors:** Lauren Hunt

**Affiliations:** University of California, San Francisco, San Francisco, California, United States

## Abstract

1 in 3 older adults die with or from dementia. The final years of life for older adults with dementia is characterized by high symptom burden, functional decline, and high costs. Hospice is the standard of care for terminally ill individuals and is known to improve end-of-life care quality and reduce costs. In recent years, hospice use has grown dramatically for older adults with dementia, who now comprise 50% of the 1.7 million hospice enrollees annually. Yet hospice was designed to address the needs of cancer patients. Additionally, the rapid growth of older adults with dementia in hospice is intertwined with increasing concerns around misuse of the Medicare Hospice Benefit. This lecture will review the history and current trends in hospice use for older adults with dementia and discuss the role of hospice and alternative care models in addressing end-of-life care needs for people with dementia and their families.

